# Complete Revascularization and Survival in STEMI

**DOI:** 10.5334/gh.1040

**Published:** 2021-09-29

**Authors:** Miha Sustersic, Miha Mrak, Polona Svegl, Anamarija Rebolj Kodre, Igor Kranjec, Zlatko Fras, Matjaz Bunc

**Affiliations:** 1 Department of Cardiology, University Medical Centre Ljubljana, Ljubljana, SI; 2 Nova Ljubljanska banka d.d., Ljubljana, SI

**Keywords:** STEMI, multivessel disease, percutaneous coronary intervention, outcomes

## Abstract

**Background:**

Complete revascularization (CR) of ST-elevation myocardial infarction patients with multivessel coronary artery disease (MVD) has proven better regarding combined endpoints than incomplete revascularization (IR) in recent randomized control trials with no impact on survival.

**Objective:**

To retrospectively evaluate the impact of complete CR during the index hospitalization on survival in STEMI patients with MVD.

**Methods and results:**

We included all patients with MVD who underwent successful primary percutaneous coronary intervention for STEMI during their index hospitalization at the University Medical Centre Ljubljana, Slovenia (from 1 January 2009 to 3 April 2011). Coronary angiograms were reviewed for non-culprit coronary arteries (>2 mm in diameter and ≥50% stenosis) treated with percutaneous coronary intervention. Rates of all-cause and cardiovascular death were compared between 235 patients who underwent CR (N = 70) or IR (N = 165). After a median follow-up of 7.0 years (interquartile range 6.0–8.2) the CR group had lower rates of all-cause death (15.7% vs 35.8%, log-rank p = 0.003) and cardiovascular death (12.9% vs 23.6%, log-rank p = 0.046). Multivariable analysis with adjustment for confounders showed no benefit of CR for all-cause death (hazard ratio [HR] 0.60, 95% confidence interval [CI] 0.31–1.18, p = 0.139) or cardiovascular death (HR 0.80, 95% CI 0.37–1.72, p = 0.560). Age, elevated serum creatinine at inclusion, diabetes and cardiogenic shock at presentation were predictors of death.

**Conclusions:**

Patients with STEMI and MVD who underwent CR showed lower all-cause and cardiovascular death during follow-up than those who underwent IR. However, after adjustment for confounders, the real determinates of survival were independent of the revascularization method.

## Background and objective

The goal of interventional cardiology in acute ST-segment elevation myocardial infarction (STEMI) is to promptly re-establish blood flow by performing primary percutaneous coronary intervention (PCI) of the occluded coronary artery, thus saving as much of the myocardium at risk as possible.

The presentation of patients with STEMI varies from almost asymptomatic to life-threatening, depending on the size of the culprit artery territory, global non-culprit coronary atherosclerotic burden, contractility of the myocardium, and comorbidities. Up to 50% of STEMI patients have significant multivessel coronary artery disease (MVD), which unfavourably affects their prognosis [1]. Evidence on whether and when PCI of non-culprit lesions should be performed is conflicting. Whereas most prospective randomized trials have shown a benefit of complete revascularization (CR) over incomplete revascularization (IR) of the culprit artery at the time of PCI or during staged PCI (i.e. during the index PCI or planned early rehospitalization) [2,3,4,5,6], one trial reported no difference [7]. Several registries have shown that CR was associated with worse outcomes versus culprit-only revascularization, both in the acute setting and over the longer term [8,9,10]. Given the paucity of long-term data, we sought to evaluate the effect of CR during index hospitalization on long-term survival in patients with STEMI and MVD treated in routine clinical practice.

## Methods

We included all STEMI patients with MVD who underwent primary PCI at the University Medical Centre Ljubljana (UMCL) Slovenia during their index hospitalization, regardless of previous hospitalizations or revascularizations. UMCL is the referring hospital for STEMI patients and treats approximately 75% of all such patients in Slovenia. Data on the index hospitalization were extracted retrospectively from patients’ medical records.

The study was approved by the Slovene medical ethics committee and was conducted following the Declaration of Helsinki. Written informed consent was not required as the data were obtained retrospectively and patient confidentiality was maintained.

Patients with a STEMI diagnosis were screened from the catheterization laboratory database. The diagnosis, according to the fourth universal definition [11], was verified using the electrocardiograms recorded at first medical contact. Patients with STEMI and MVD who underwent successful PCI were included. To ensure representativeness, patients with cardiogenic shock, left-main stem disease, chronic total occlusions (CTO), and previous myocardial infarction or coronary artery bypass grafting (CABG) were included. All patients were treated for ≥24 hours in the coronary care unit, after which they were discharged to a cardiology ward or transferred to a regional hospital.

During the period when the patients were treated, all STEMI patients in Slovenia routinely received unfractionated heparin (100 U/kg) and dual antiplatelet therapy with a loading dose (aspirin 250–500 mg and clopidogrel 600 mg), before or at the time of PCI. The femoral artery was the predominant access site. PCI was done with or without pre-dilatation, thromboaspiration, direct stenting (bare metal or drug-eluting), or balloon angioplasty, at the interventional cardiologist’s discretion. PCI of the culprit artery was declared successful if Thrombolysis In Myocardial Infarction (TIMI) flow grade was ≥2.

Before inclusion in the study, all coronary angiograms done during the index hospitalization were reviewed by two experienced PCI operators, who had no insights into further treatment, to determine the presence of MVD, stenosis number and severity. To fulfil the MVD criteria, at least one of the non-culprit coronary arteries (>2 mm in diameter and ≥50% stenosis) had to be affected. In case of discrepancies in the estimated severity of non-culprit stenosis, the angiograms were re-reviewed and consensus reached. The residual SYNTAX Score I (Synergy Between PCI With Taxus and Cardiac Surgery) was calculated after PCI of the culprit lesion. Non-culprit lesions had to be treated medically or with PCI during the index hospitalization.

Patients were divided according to whether they underwent CR or IR during the index hospitalization. CR was defined as revascularization performed during the index PCI or delayed PCI done during the same hospitalization. Study outcomes were all-cause death and cardiovascular death. Cardiovascular death was defined as fatal acute myocardial infarction, sudden cardiac death, death due to heart failure, stroke, cardiovascular procedures, cardiovascular haemorrhage or other cardiovascular causes [12]. Follow-up and mortality data were recorded up to 1 April 2017. Mortality data were obtained from the Slovene National Institute of Public Health.

Data on risk factors and cardiovascular comorbidities were collected from discharge documents and blood samples. Blood was sampled before or at the time of the PCI, as well as subsequently, at the physician’s discretion. Data were also collected on the highest serum troponin I ultra and creatinine concentrations, lowest blood haemoglobin value, and lipid status. Echocardiographic assessment of left ventricular ejection fraction (LVEF) was performed by Simpson’s method before release/transfer. All echocardiographic data were obtained from the echocardiographic examinations and were reviewed by an echocardiographer who was blinded to the study outcomes and individual coronary anatomy. Data on medications were obtained from patients’ medical records at the time of the index admission and discharge or transfer.

### Statistical analysis

Information on the sample size estimation is included in the Supplementary Appendix. Descriptive statistics are reported as mean±standard deviation (SD) and count (percentage). Comparison between groups was tested using the Mann-Whitney or Fisher’s exact test. Non-parametric survival analysis was performed with the Kaplan-Meier method. Survival distributions of two or more independent groups were compared using the log-rank test. A Cox proportional hazards model was used to evaluate the effect of several factors on clinical outcomes. An adjusted Cox proportional hazards model was used to rule out potential confounders (age, diabetes, chronic kidney disease, previous myocardial infarction or percutaneous revascularization, cardiogenic shock, CTO, residual SYNTAX I score, and rehospitalization for the acute coronary syndrome, stable angina or heart failure). All hypotheses were tested at a prespecified significance level of <0.05. Data analysis and statistics were performed using software package R (version 4.0, R Foundation for Statistical Computing, Vienna, Austria).

## Results

Between 1 January 2009 and 3 April 2011, we identified 810 patients with STEMI, of which 258 (31.9%) had MVD. Twenty-three patients (9%) were excluded. The study population therefore comprised 235 (91%) patients: 70 (30%) underwent CR and 165 (70%) IR (Supplementary Figure).

Demographic data, comorbidities, chronic therapies, and LVEF after intended PCI were similar in the two groups (Table 1, Supplementary Table 1). The IR group had a greater burden of non-culprit coronary artery stenosis (p = 0.005) and CTO (p < 0.001); the CR group underwent more coronary interventions during the index hospitalization (p < 0.001) (Table 1). There were no differences between groups concerning laboratory parameters (Supplementary Table 1). Patients in the IR group had numerically, but not statistically significantly, higher creatinine levels at the time of the procedure; there were no differences between groups in the highest creatinine values after PCI had been performed. At the time of discharge/transfer, use of beta-blockers and statins was lower in the IR group (p = 0.031 and p = 0.004, respectively).

**Table 1:** Patient and procedural characteristics.

Variable	CR N = 70	IR N = 165	*p*-value

Age ≥61 years, n (%)	41 (59)	114 (69)	0.160
Men, n (%)	49 (70)	120 (73)	0.790
Arterial hypertension, n (%)	43 (61)	116 (70)	0.239
Diabetes, n (%)	11 (16)	42 (25)	0.143
Current smoker, n (%)	26 (37)	57 (35)	0.817
Hyperlipidaemia, n (%)	46 (66)	104 (63)	0.808
Family history of cardiovascular disease*, n (%)	11 (16)	30 (18)	0.789
Chronic kidney disease, n (%)	6 (9)	23 (14)	0.354
Previous myocardial infarction, n (%)	4 (6)	23 (14)	0.077
Previous PCI, n (%)	5 (7)	13 (8)	1.000
Previous CABG, n (%)	0 (0)	6 (4)	0.183
Coronary intervention			
Culprit artery			
Left descending coronary	26 (37)	57 (35)	0.766
Right coronary	36 (51)	81 (49)	0.424
Left circumflex coronary	8 (11)	27 (16)	0.777
Number of significant stenoses of non-culprit artery			**0.005**
1	38 (54)	56 (34)	
>1	32 (46)	109 (66)	
CTO	1 (1)	29 (18)	**<0.001**
After CABG		4 (2)	0.321
Number of PCI procedures^†^			
1	32 (46)	130 (79)	**<0.001**
>1	38 (54)	35 (21)	**<0.001**
Integrilin use	13 (19)	34 (21)	0.859
Intra-aortic balloon pump	5 (7)	26 (16)	0.092
Transfusion due to coronary intervention complication	4 (6)	8 (5)	0.754
LVEF after PCI^†^			0.871
>55%	20 (29)	40 (24)	
45 to ≤54%	7 (10)	21 (13)	
30 to <45%	7 (10)	13 (8)	
<30%	3 (4)	10 (6)	

* <55 years in men and <65 years in women; ^†^ During index hospitalization. CABG: coronary artery bypass graft; CR: complete revascularization; CTO: chronic total occlusion; IR: incomplete revascularization; LVEF: left ventricular ejection fraction; PCI: percutaneous coronary intervention; TIMI: Thrombolysis In Myocardial Infarction flow.

The severity of non-culprit stenosis was higher in the CR group (p = 0.015), whereas residual SYNTAX score I was higher in the IR group (p = 0.011) (Supplementary Table 2). Bare-metal versus drug-eluting stents were more frequently used in both groups.

### All-cause death

**Figure 1:**
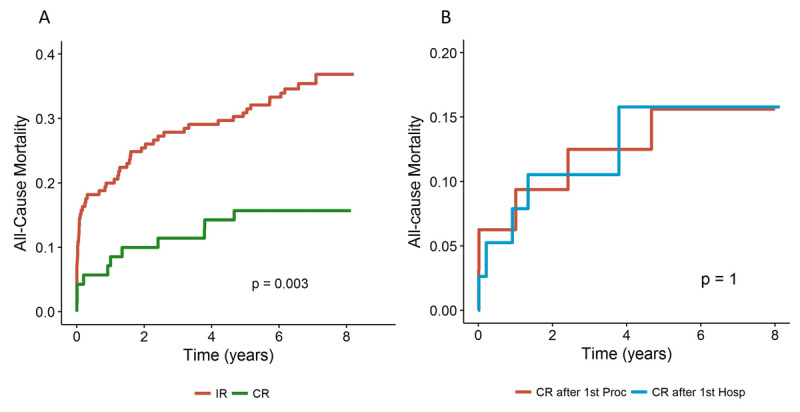
All-cause death: **(A)** comparing CR and IR during the index hospitalization; **(B)** according to CR during index procedure or staged PCI. CR: complete revascularization; IR: incomplete revascularization.

All-cause death occurred in 11 patients (15.7%) in the CR group (95% CI for mean time to death 0.49–2.81 years) and in 59 patients (35.8%) in the IR group (95% CI for mean time to death 1.04–2.15 years; log-rank p = 0.003) (Figure 1A). Risk of all-cause death was 2.6 times lower in the CR group (HR 0.39, 95% CI 0.20–0.74, p = 0.004). There was no difference in overall survival between patients who underwent CR during the index procedure and those who had staged PCI (Figure 1B).

**Figure 2:**
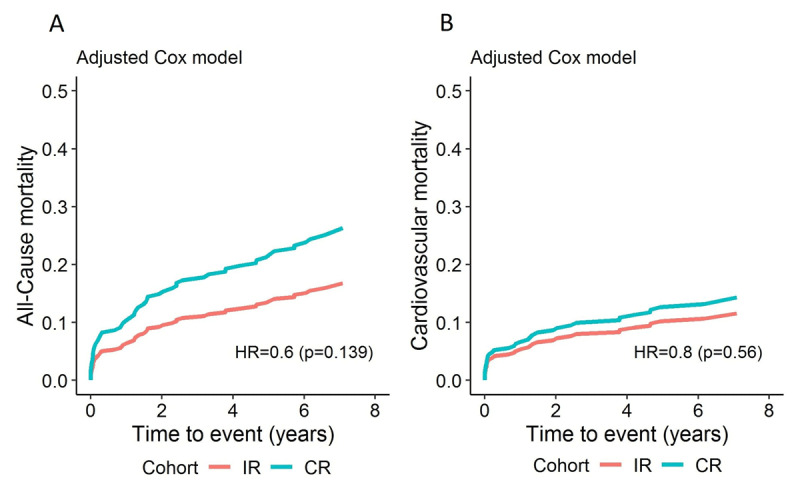
Adjusted* Cox proportional hazard models, comparing CR and IR, for **(A)** all-cause death; and **(B)** cardiovascular death. CR: complete revascularization; IR: incomplete revascularization. *Adjusted for age, diabetes, chronic kidney disease, previous myocardial infarction, previous percutaneous revascularization, cardiogenic shock, presence of chronic total occlusion, residual SYNTAX I score, and rehospitalization.

In a stratified Cox model, all-cause death was increased in male patients and in those with arterial hypertension, diabetes, hyperlipidaemia, haemoglobin <120 g/L or creatinine >97 µmol/L before the index intervention, and LVEF <55%. IR in the absence of CTO, one significant coronary lesion left untreated with PCI, and a single PCI also led to an increased risk of all-cause death (Supplementary Table 3). Several factors, excluding previous myocardial infarction or PCI and CTO, significantly affected the rate of all-cause death in comparison to those who were at the end of follow-up (Supplementary Table 4). In the adjusted Cox proportional hazards model, CR was no longer significant for all-cause death (Figure 2A). The HR for all-cause death was higher with age (p < 0.001), diabetes (p = 0.04), elevated serum creatinine value before the index event (p = 0.001), and cardiogenic shock at presentation (p < 0.001) (Table 2).

**Table 2:** Independent predictors of all-cause and cardiovascular death with CR versus IR (multivariable Cox model*).

Predictor	All-cause death			Cardiovascular death		

	Coefficient	HR (95% CI)	*p*-value	Coefficient	HR (95% CI)	*p*-value

CR	–0.51	0.60 (0.31–1.18)	0.139	–0.23	0.80 (0.37–1.72)	0.560
Age	0.07	1.07 (1.04–1.10)	**<0.001**	0.08	1.08 (1.05–1.12)	**<0.001**
Diabetes	0.54	1.72 (1.02–2.91)	**0.042**	0.46	1.58 (0.83–3.03)	0.166
Creatinine value before index event (natural logarithm)	0.79	2.19 (1.39–3.46)	**0.001**	0.71	2.02 (1.13–3.64)	**0.018**
Previous myocardial infarction	0.09	1.09 (0.36–3.27)	0.875	0.78	2.18 (0.66–7.20)	0.200
Previous PCI	–0.55	0.58 (0.15–2.15)	0.411	–1.19	0.30 (0.07–1.34)	0.117
Cardiogenic shock at presentation	2.17	8.80 (3.55–21.82)	**<0.001**	2.12	8.36 (3.28–21.32)	**<0.001**
CTO	0.13	1.13 (0.57–2.25)	0.721	–0.15	0.86 (0.35–2.13)	0.744
Residual SYNTAX I score	0.01	1.01 (0.97–1.05)	0.595	0.01	1.01 (0.96–1.06)	0.681
Rehospitalization^†^	–0.56	0.57 (0.26–1.24)	0.157	–0.98	0.38 (0.13–1.10)	0.074

* Adjusted for age, diabetes, chronic kidney disease, previous myocardial infarction or percutaneous revascularization, cardiogenic shock at presentation: the need for intra-aortic balloon pump ≤15 days after inclusion, CTO, residual SYNTAX I score, and rehospitalization; ^†^ To cardiology department due to residual myocardial ischemia or acute coronary syndrome. CI: confidence interval: CR, complete revascularization; HR: hazard ratio; IABP, intra-aortic balloon pump; IR, incomplete revascularization; PCI: percutaneous coronary intervention; SYNTAX I: Synergy Between PCI With Taxus and Cardiac Surgery score I.

### Cardiovascular death

**Figure 3:**
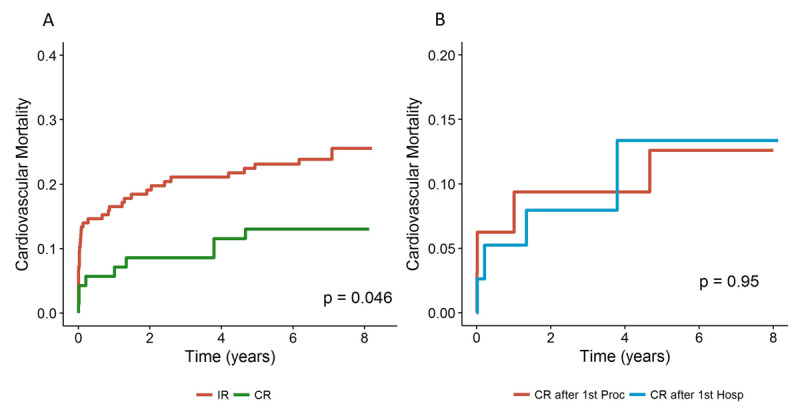
Cardiovascular death: **(A)** comparing CR and IR during the index hospitalization; **(B)** in CR group according to CR during index procedure or staged PCI. CR: complete revascularization; IR: incomplete revascularization.

Cardiovascular death occurred in nine patients (12.9%) in the CR group (95% CI for mean time to death 0.19–3.12 years) and in 39 patients (23.9%) in the IR group (95% CI for mean time to death 0.50–1.71 years; log-rank p = 0.046) (Figure 3A). The risk of cardiovascular death was 2.1 times lower in the CR group but was not statistically significantly different (HR 0.49, 95% CI 0.24–1.00; p = 0.051). In the CR group, there was no difference in cardiovascular survival between patients who underwent CR during the index procedure and those who had staged PCI (Figure 3B).

In a stratified Cox model, only severe stenosis of one non-culprit coronary artery left untreated was associated with an increased risk of cardiovascular death (Supplementary Table 3). Several factors significantly affected the rate of cardiovascular death in comparison to those alive at the end of follow up; previous myocardial infarction or PCI, CTO and residual SYNTAX I score were not predictors (Supplementary Table 4). After adjustment, no benefit for cardiovascular death was found for CR (Table 2; Figure 2B). The risk of cardiovascular death increased with age (p < 0.001), elevated serum creatinine value before the index event (p = 0.001), and cardiogenic shock at presentation (p < 0.001) (Table 2).

### Complications due to coronary interventions

The rate of serious complications due to the coronary intervention was 0.04% in the CR group and 0.09% in the IR group. All of the complications occurred during PCI of the culprit lesion in the CR group, whereas only one complication occurred during PCI of the non-culprit lesion in the IR group. There was no statistically significant difference in the occurrence of complications between groups (Supplementary Table 6).

## Discussion

The findings from this retrospective all-comers study of patients with STEMI and MVD are threefold: (1) long-term all-cause and cardiovascular death rates were lower in patients who underwent CR versus IR during the index hospitalization; (2) several clinical and angiographic variables affected all-cause death, while only severe non-culprit lesion left untreated affected cardiovascular death; and (3) the benefits of CR disappeared after adjustment for potential confounders, with the strongest independent predictors of the death being age, diabetes, higher than normal serum creatinine concentration, and cardiogenic shock at presentation.

The optimal strategy for treating non-culprit disease in ‘real-life’ STEMI patients in terms of a benefit in ‘hard’ outcomes is unclear, with no prospective studies specifically addressing this issue. Registry studies comparing CR with IR in STEMI with MVD are heterogeneous and report conflicting results [9,10,13,14,15]. Whereas one randomized trial reported no benefit with CR [7], other trials have shown better outcomes, but they included ‘soft’ outcomes (e.g. refractory angina, proven ischaemia, and repeat revascularization) in the composite endpoints [2,3,6]. A meta-analysis that focused on all-cause and cardiovascular death found that CR may be better than IR [16], but the quality of evidence on which this was based was poor and the COMPLETE and COMPARE ACUTE trials were not included [4,5]. COMPLETE is the largest prospective study conducted to date and showed that CR is better than IR for the composite outcome of cardiovascular death or myocardial infarction (HR 0.74, 95% CI 0.60–0.91; p = 0.004), driven primarily by a reduction in myocardial infarction [4]. The benefit of CR was consistent regardless of whether non-culprit-lesion PCI was performed during the index hospitalization or shortly after discharge. In our analysis, which differentiated CR from IR during the index hospitalization, we found no difference in outcomes for CR whether it was accomplished during the index procedure or with staged PCI.

In the National Cardiovascular Data Registry, compared with the IR group, patients in the CR group were younger, had less complex coronary disease, and had a higher prevalence of cardiogenic shock [8]. Populations in randomized trials are highly selected, and patients with the severe left-main disease, CTO, cardiogenic shock, previous coronary artery bypass, or who are planned for revascularization are largely excluded from STEMI trials. In the present study, whereas the baseline characteristics of the two groups were largely similar, the IR group had a higher burden of stenosis, higher residual SYNTAX score, and more CTOs, which probably led to fewer PCIs during the index hospitalization. This may also explain the similar number of stents used per patient in both groups. Besides, the IR group had numerically higher use of intra-aortic balloon pumps during the index intervention, reflecting cardiogenic shock, which did not influence the LVEF difference before discharge or transfer. Use of beta-blockers and statins also differed between groups but may have changed following discharge or transfer. Among several clinical risk factors, the risk for all-cause death was increased in the IR group due to two angiographic characteristics (only one severe non-culprit stenosis left untreated and IR in the absence of CTO) whereas cardiovascular mortality risk was increased only when the severe non-culprit stenosis was left untreated.

Estimation of the severity of non-culprit lesions and the definition of ischaemic territory may also pose problems in patients with STEMI and MVD. Prospective trials have used either angiographic stenosis estimation or invasive functional assessment [2,3,4,5,6], but neither are perfect because vasoconstriction is increased during the acute stage and lesions could be overestimated visually and functionally [17]. Whereas severe stenosis may not always cause significant ischaemia to justify intervention, stenosis slightly >50% may cause ischaemia in large coronary beds [18]. Even passing the wire through non-culprit lesion may be deleterious in patients with the acute coronary syndrome. Future studies (e.g. iMODERN, NCT03298659) are needed to investigate the accuracy of functional assessments in STEMI and when they should be performed. In our study, we used visual angiographic estimation of non-culprit lesion severity and most lesions were >70%, but we did not perform a functional assessment of lesion severity, and the degree of stenosis may be irrelevant if the vessel involves a small territory or the myocardium is not viable.

Intervention on non-culprit coronary arteries can lead to complications [19,20]. However, postponing an intervention necessitates using another arterial access site, which can cause complications at the puncture site. In the present study, the rate of serious complications due to PCI did not differ between CR and IR groups. Some IR patients may not have been completely revascularized due to complications during the index intervention, as all but one complication occurred during PCI of the culprit lesion. During the study period, we used only the femoral access site, whereas the radial access site has a lower complication rate and is now the preferred access site [21].

The main strengths of this study are the long follow-up, inclusion of patients treated in routine clinical practice, and choice of hard outcomes. This was, however, a single-centre study with a relatively small number of patients. The sample size was sufficient to address all-cause but not cardiovascular death. As a retrospective analysis, it may be subject to selection bias, missing data and under-reporting of events; the effects of residual confounding also cannot be excluded. Data on changes in medical therapies after discharge or transfer were not collected. We could not measure the severity of ischaemia of non-culprit lesions, and PCI was guided only by the severity of non-culprit stenosis.

## Conclusions

Patients with STEMI and MVD treated with CR during the index hospitalization had lower long-term rates of all-cause and lower cardiovascular death compared to patients who underwent IR during the index hospitalization, but this benefit disappeared after adjustment for confounders. Advanced age, diabetes, elevated serum creatinine, and cardiogenic shock at presentation were, however, associated with death. The treatment of STEMI patients with MVD should follow a careful patient-tailored approach. Which patients with STEMI and MVD may truly have a survival benefit from CR will have to be determined in an adequately powered randomized trial.

## Impact on daily practice

The results of this retrospective study suggest that complete versus incomplete revascularization during the index admission in patients with STEMI and MVD disease does not improve long-term survival. Optimal management of clinical risk factors may be more beneficial in improving prognosis in this population.

## Additional File

The additional file for this article can be found as follows:

10.5334/gh.1040.s1 Supplementary Appendix. Appendices and online data supplement legends.

## Figures and Tables

**Table:** 

CABG	Coronary Artery Bypass Graft
CR	Complete Revascularization
CTO	Chronic Total Occlusion
IR	Incomplete Revascularization
LVEF	Left Ventricular Ejection Fraction
MVD	Multivessel Coronary Artery Disease
PCI	Percutaneous Coronary Intervention
STEMI	ST-segment Elevation Myocardial Infarction
SYNTAX	Synergy Between PCI With Taxus and Cardiac Surgery
TIMI	Thrombolysis In Myocardial Infarction
UMCL	University Medical Centre Ljubljana
