# Correction: Complete Revascularization and Survival in STEMI

**DOI:** 10.5334/gh.1080

**Published:** 2021-11-03

**Authors:** Miha Sustersic, Miha Mrak, Polona Svegl, Anamarija Rebolj Kodre, Igor Kranjec, Zlatko Fras, Matjaz Bunc

**Affiliations:** 1 Department of Cardiology, University Medical Centre Ljubljana, Ljubljana, SI; 2 Nova Ljubljanska banka d.d., Ljubljana, SI

**Keywords:** STEMI, multivessel disease, percutaneous coronary intervention, outcomes

## Abstract

This article details a correction to: Sustersic M, Mrak M, Svegl P, Kodre AR, Kranjec I, Fras Z, et al. Complete Revascularization and Survival in STEMI. *Global Heart*. 2021; 16(1): 64. DOI: http://doi.org/10.5334/gh.1040

## Correction

It has come to the authors’ attention that there is an error in (http://doi.org/10.5334/gh.1040) [1] which needs to be corrected. In the ‘*Complications due to coronary interventions*’ section, it is stated that:

‘The rate of serious complications due to the coronary intervention was 0.04% in the CR group and 0.09% in the IR group.’

However, 0.04 and 0.09 are quotients and not percentages. Instead, as stated correctly in Supplementary Table 6 of the original article, it should be written:

‘The rate of serious complications due to the coronary intervention was 4.3% in the CR group and 9.7% in the IR group.’
