# Supplementary material for: Complete Revascularization and Survival in STEMI
Source: Glob Heart. 2021 Sep 29;16(1):64. doi: 10.5334/gh.1040 (PMC8485869; doi:10.5334/gh.1040)
Supplement: Supplementary Appendix. — Appendices and online data supplement legends. [file gh-16-1-1040-s1.pdf]

Supplementary Appendix

Contents

Additional information on the study methods 2

Supplementary Table 1. Cardiovascular therapies and laboratory variables. 3

Supplementary Table 2. The severity of non-culprit stenosis, residual SYNTAX I score, and coronary stent characteristics. 5

Supplementary Table 3. Stratified Cox model for all-cause and cardiovascular death comparing IR with CR. 6

Supplementary Table 4. Distribution of all-cause and cardiovascular death rates in comparison to those alive per selected risk factor (categorical variables) in all patients, CR, and IR group. 8

Supplementary Table 5. Distribution of all-cause and cardiovascular death rates in comparison to those alive per selected risk factor (numerical variables) in all patients, CR, and IR group. 10

Supplementary Table 6. Complications due to the coronary intervention 11

Supplementary Figure. *Study flow chart.* 12

References 13

Additional information on the study methods

Based on retrospective experience at University Medical Centre Ljubljana (UMCL) Slovenia, approximately 30% of patients with ST-segment elevation myocardial infarction (STEMI) and multivessel coronary artery disease (MVD) would undergo complete revascularization (CR) during their index hospitalization. In registry studies, the reported incidence of 1-year death in STEMI is approximately 10% and is two- to threefold higher in STEMI with MVD[1,2]. We, therefore, assumed that patients with CR would have a 1-year death rate closer to patients with STEMI and single-vessel coronary artery disease (approximately 10%), whereas the incidence of death would be 2.5 times higher (approximately 25%) in patients with incomplete revascularization. Most deaths would be cardiovascular. According to the incidence of STEMI patients treated with a percutaneous coronary intervention (PCI) in our hospital, we calculated that with an alpha of 0.05 and a power of 80%, at least 248 patients would have to be enrolled to gain adequate statistical power to see a mortality difference. Therefore, we calculated that the length of enrolment would have to be ≥2.3 years. Enrolment started on 1 January 2009. Follow-up continued until 1 April 2017, to obtain mortality data for ≥6 years of follow-up.

Supplementary Table 1. Cardiovascular therapies and laboratory variables.

**Variable**	**CR**<br>**N=70**	**IR**<br>**N=165**	***p*-value**
Therapy before admission, n (%)			
Antiplatelet (aspirin or clopidogrel)	0 (0%)	4 (2.4%)	0.321
Anticoagulant (warfarin or non-vitamin K antagonist oral anticoagulant)	9 (12.9%)	27 (16.4%)	0.558
Dual antiplatelet	1 (1.4%)	1 (0.6%)	0.508
Beta-blocker	12(17.1%)	33 (20%)	0.718
Calcium channel blocker	8 (11.4%)	27 (16.4%)	0.424
Angiotensin-converting enzyme inhibitor	16 (22.9%)	55 (33.3%)	0.122
Angiotensin receptor blocker	7 (10.0%)	19 (11.5%)	0.823
Mineralocorticoid receptor blocker	0 (0%)	3 (1.8%)	0.556
Statin	13 (18.6%)	39 (23.6%)	0.492
Ezetimibe	1 (1.4%)	1 (0.6%)	0.508
Therapy at discharge/transfer to regional hospital, n (%)			
Aspirin	70 (100%)	165 (100%)	1.000
Clopidogrel	67 (95.7%)	149 (90.3%)	0.199
Prasugrel	3 (4.3%)	7 (4.2%)	1.000
Beta-blocker	47 (67.1%)	85 (51.5%)	**0.031**
Calcium-channel blocker	4 (5.7%)	8 (4.8%)	0.754
Angiotensin-converting enzyme inhibitor	57 (81.4%)	113 (58.6%)	0.055
Angiotensin receptor blocker	5 (7.1%)	4 (2.4%)	0.131
Mineralocorticoid receptor blocker	9 (12.9%)	16 (9.7%)	0.492
Statin	68 (97.1%)	138 (83.6%)	**0.004**
Ezetimibe	3 (4.3%)	3 (1.8%)	0.366
Laboratory variables, mean±SD			
Maximum troponin I ultra value during index hospitalization (µg/L)	(n=60)<br>72.7±80	(n=150)<br>99±115.6	0.385
Low-density lipoprotein cholesterol (mmol/L)	(n=40)<br>3.3±1.3	(n=86)<br>3.2±1.2	0.975
Blood hemoglobin (g/L)			
Before or at index intervention	(n=56)<br>130.1±18.5	(n=129)<br>129.9±17.4	0.702
Lowest concentration during index hospitalization	(n=44)<br>126.5±19.3	(n=99)<br>127.1±15.8	0.894
Blood creatinine (µmol/L)			
Before or at index intervention	(n=56)<br>84.0±26.0	(n=135)<br>112.5±95	0.078

**Variable**	**CR N=70**	**IR N=165**	***p*-value**
Highest concentration after PCI during index hospitalization	(n=57) 87.6±51.1	(n=123) 105±87.8	0.487
CR: complete revascularization; IR: incomplete revascularization; PCI: percutaneous coronary intervention.			

Supplementary Table 2. The severity of non-culprit stenosis, residual SYNTAX I score, and coronary stent characteristics.

	CR group (N=70)			IR group (N=165)			*p*-value
**Variable**	**n**	**Mean±SD**	**Median (Q1, Q3)**	**n**	**Mean±SD**	**Median (Q1, Q3)**	
Residual SYNTAX I score	70	6.4±4.5	5 (2.3, 7.8)	165	8.7±6.4	7.0 (4.0, 13.0)	0.011
Non-culprit stenosis severity (%)	70	83.6±12.3	85.0 (80.0, 95.0)	165	79.7±13.4	78.3 (70.0, 90.0)	0.015
Number of stents implanted per patient							
Drug eluting	32	1.8±1.0	2 (1.0, 2.0)	37	2.0±1.1	2 (1.0, 2.0)	0.667
Bare metal	62	2.4±1.4	2 (1.0, 3.0)	129	2.1±1.3	2 (1.0, 3.0)	0.057

CR: complete revascularization; IR: incomplete revascularization; Q: quartile; SD: standard deviation; SYNTAX I: Synergy Between PCI With Taxus and Cardiac Surgery score I.

Supplementary Table 3. Stratified Cox model for all-cause and cardiovascular death comparing IR with CR.

	All-cause death			Cardiovascular death		
**Risk factor**	**Coefficient**	**HR (95% CI)**	***p*-value**	**Coefficient**	**HR (95% CI)**	***p*-value**
Age						
≤65 years	1.29	3.64 (0.81−16.42)	0.093	0.49	1.64 (0.32−8.44)	0.556
>65 years	0.60	1.81 (0.89−3.70)	0.102	0.48	1.62 (0.72–3.66)	0.243
Sex						
Male	1.14	3.12 (1.22−7.98)	**0.018**	0.65	1.91 (0.64–5.7)	0.244
Female	0.85	2.34 (0.96−5.71)	0.062	0.89	2.44 (0.92–6.44)	0.073
Arterial hypertension	1.21	3.36 (1.44−7.87)	**0.005**	0.91	2.47 (0.96–6.39)	0.062
Diabetes	2.17	8.74 (1.18−64.70)	**0.034**	19.50	−	0.998*
Smoker	1.10	2.99 (0.67−13.38)	0.151	0.55	1.72 (0.36–8.30)	0.497
Hyperlipidaemia	1.15	3.16 (1.23−8.11)	**0.017**	0.55	1.74 (0.64–4.69)	0.274
Chronic kidney disease	1.96	7.09 (0.94−53.49)	0.058	1.49	4.43 (0.57−34.18)	0.154
Maximum troponin value* <100 µg/L	0.44	1.55 (0.73−3.27)	0.252	0.09	1.09 (0.45–2.65)	0.846
LDL-C >3 mmol/L	1.09	2.98 (0.67−13.21)	0.150	0.60	1.82 (0.39–8.57)	0.449
Hemoglobin <120 g/L						
Before PCI	1.18	3.26 (1.24−8.56)	**0.016**	0.81	2.24 (0.74–6.80)	0.154
After PCI	0.93	2.54 (0.85−7.56)	0.094	0.45	1.57 (0.41-5.92)	0.509
Creatinine >97 µmol/L						
Before PCI	1.28	3.61 (1.10−11.83)	**0.034**	1.17	3.22 (0.75–13.86)	0.116
After PCI	0.75	2.12 (0.73−6.17)	0.170	0.75	2.12 (0.62–7.30)	0.231
Culprit coronary artery						
LAD	0.87	2.40 (0.99−5.83)	0.054	0.72	2.06 (0.72–2.06)	0.150
LCX	1.36	3.88 (0.50−30.23)	0.196	1.16	3.18 (1.16–3.18)	0.274
RCA	0.97	2.64 (0.91−7.66)	0.074	0.55	1.73 (0.55–1.73)	0.398
Number of non-culprit significant stenoses						
1	1.53	4.63 (1.36−15.72)	**0.014**	2.21	9.12 (2.21–9.12)	**0.034**
>1	0.51	1.67 (0.78−3.56)	0.186	0.08	1.08 (0.08–1.08)	0.846
No CTO	0.91	2.50	**0.006**	0.73	2.07	0.054

		(1.30−4.81)			(0.73–2.07)	
Number of PCI procedures*						
1	1.01	2.75 (1.09−6.89)	**0.032**	0.72	2.06 (0.72–2.06)	0.176
>1	0.73	2.07 (0.75−5.69)	0.160	0.79	2.21 (0.79–2.21)	0.155
Transfusion†	1.17	3.23 (0.38−27.72)	0.286	0.64	1.89 (0.64–1.89)	0.583
Left ventricular ejection fraction						
≥55%	0.48	1.62 (0.44−5.98)	0.471	0.23	1.26 (0.23–1.26)	0.738
<55%	1.28	3.61 (1.08−12.09)	**0.037**	1.41	4.10 (1.41–4.10)	0.059

*during index hospitalization; due to coronary intervention complication. CI: confidence interval; CR: complete revascularization; HR: hazard ratio; IR: incomplete revascularization; SD: standard deviation; SYNTAX I: Synergy Between PCI With Taxus and Cardiac Surgery score I.

Supplementary Table 4. Distribution of all-cause and cardiovascular death rates in comparison to those alive per selected risk factor (categorical variables) in all patients, CR, and IR group.

	All patients (N=235)				CR (N=70)				IR (N=165)			
**Variable**	**N (%)**	**Deaths (%)**	**95% CI**	***p*-value**	**N (%)**	**Deaths (%)**	**95% CI**	***p*-value**	**N (%)**	**Deaths (%)**	**95% CI**	***p*-value**
All-cause death												
Male sex	169 (71.9)	39 (23.1)	17–30.2	<0.001	49 (70)	5 (10.2)	3.4–22.2	0.075	120 (72.7)	34 (28.3)	20.5–37.3	0.002
Diabetes	53 (22.6)	25 (47.2)	33.3–61.4	0.003	11 (15.7)	1 (9.1)	0.2–41.3	1.000	42 (25.5)	24 (57.1)	41–72.3	0.001
Chronic kidney disease	29 (12.3)	18 (62.1)	42.3–79.3	<0.001	6 (8.6)	1 (16.7)	0.4–64.1	1.000	23 (13.9)	17 (73.9)	51.6–89.8	<0.001
Creatinine >97 µmol/L before index event	58 (24.7)	33 (56.9)	43.2–69.8	<0.001	12 (17.1)	3 (25)	5.5–57.2	0.386	46 (27.9)	30 (65.2)	49.8–78.6	<0.001
Creatinine >97 µmol/L after revascularization	48 (20.4)	25 (52.1)	37.2–66.7	<0.001	12 (17.1)	4 (33.3)	9.9–65.1	0.086	36 (21.8)	21 (58.3)	40.8–74.5	0.003
Previous myocardial infarction	27 (11.5)	10 (37)	19.4–57.6	0.379	4 (5.7)	0 (0)	0–60.2	1.000	23 (13.9)	10 (43.5)	23.2–65.5	0.483
Previous PCI	18 (7.7)	6 (33.3)	13.3–59	0.790	5 (7.1)	0 (0)	0–52.2	1.000	13 (7.9)	6 (46.2)	19.2–74.9	0.548
Cardiogenic shock*	31 (13.2)	18 (58.1)	39.1–75.5	0.001	5 (7.1)	3 (60)	14.7–94.7	0.025	26 (15.8)	15 (57.7)	36.9–76.6	0.014
Presence of chronic total occlusions	30 (12.8)	11 (36.7)	19.9–56.1	0.379	1 (1.4)	0 (0)	0–97.5	1.000	29 (17.6)	11 (37.9)	20.7–57.7	0.832
Rehospitalization	51 (21.7)	9 (17.6)	8.4–30.9	0.038	14 (20)	2 (14.3)	1.8–42.8	1.000	37 (22.4)	7 (18.9)	8–35.2	0.019
Cardiovascular death												
Male sex	169 (71.9)	21 (12.4)	7.9–18.4	<0.001	49 (70)	4 (8.2)	2.3–19.6	0.116	120 (72.7)	17 (14.2)	8.5–21.7	<0.001
Diabetes	53 (22.6)	17 (32.1)	19.9–46.3	0.021	11 (15.7)	0 (0)	0–28.5	0.336	42 (25.5)	17 (40.5)	25.6–56.7	0.006
Chronic kidney disease	29 (12.3)	13 (44.8)	26.4–64.3	0.002	6 (8.6)	1 (16.7)	0.4–64.1	0.577	23 (13.9)	12 (52.2)	30.6–73.2	0.001

Creatinine >97 µmol/L before index event	58 (24.7)	21 (36.2)	24–49.9	0.001	12 (17.1)	2 (16.7)	2.1–48.4	0.646	46 (27.9)	19 (41.3)	27–56.8	0.002
Creatinine >97 µmol/L after revascularization	48 (20.4)	19 (39.6)	25.8–54.7	0.001	12 (17.1)	3 (25)	5.5–57.2	0.177	36 (21.8)	16 (44.4)	27.9–61.9	0.002
Previous myocardial infarction	27 (11.5)	8 (29.6)	13.8–50.2	0.211	4 (5.7)	0 (0)	0–60.2	1.000	23 (13.9)	8 (34.8)	16.4–57.3	0.191
Previous PCI	18 (7.7)	4 (22.2)	6.4–47.6	0.768	5 (7.1)	0 (0)	0–52.2	1.000	13 (7.9)	4 (30.8)	9.1–61.4	0.508
Cardiogenic shock*	31 (13.2)	16 (51.6)	33.1–69.8	<0.001	5 (7.1)	3 (60)	14.7–94.7	0.013	26 (15.8)	13 (50)	29.9–70.1	0.002
Presence of chronic total occlusions	30 (12.8)	6 (20)	7.7–38.6	1.000	1 (1.4)	0 (0)	0–97.5	1.000	29 (17.6)	6 (20.7)	8–39.7	0.812
Rehospitalization	51 (21.7)	5 (9.8)	3.3–21.4	0.032	14 (20.0)	1 (7.1)	0.2–33.9	0.675	37 (22.4)	4 (10.8)	3–25.4	0.047

*the need for Intra-aortic balloon pump in first 15 days of STEMI. CI: confidence interval; CR: complete revascularization; IR: incomplete revascularization.

Supplementary Table 5. Distribution of all-cause and cardiovascular death rates in comparison to those alive per selected risk factor (numerical variables) in all patients, CR, and IR group.

	**All patients (N=235)**			**CR (N=70)**			**IR (N=165)**		
	**Alive**	**Dead**	***p*-value**	**Alive**	**Dead**	***p*-value**	**Alive**	**Dead**	***p*-value**
All-cause death									
Age, years (mean ± SD)	63.9 ± 11.1	75.5 ± 10.2	<0.001	62.6 ± 11.2	75.7 ± 8.3	0.001	64.6 ± 11.0	75.5 ± 10.5	<0.001
ln*(CR before) (mean ± SD)	3.52 ± 1.79	4.02 ± 1.81	<0.001	3.48 ± 1.79	3.68 ± 1.84	0.395	3.54 ± 1.80	4.08 ± 1.81	<0.001
Residual SYNTAX score (mean ± SD)	7.41 ± 5.67	9.34 ± 6.56	0.021	6.36 ± 4.83	6.45 ± 3.05	0.449	7.99 ± 6.03	9.88 ± 6.91	0.075
Cardiovascular death									
Age, years (mean ± SD)	64.9 ± 11.1	77.0 ± 10.5	<0.001	63.1 ± 11.3	75.7 ± 9.1	0.003	65.8 ± 11.0	77.3 ± 10.9	<0.001
ln*(CR before) (mean ± SD)	3.61 ± 1.77	3.88 ± 1.94	0.005	3.52 ± 1.77	3.47 ± 1.99	0.744	3.66 ± 1.78	3.97 ± 1.95	0.005
Residual SYNTAX score (mean ± SD)	7.61 ± 5.61	9.42 ± 7.23	0.164	6.36 ± 4.75	6.44 ± 3.4	0.589	8.22 ± 5.9	10.1 ± 7.73	0.299

*ln: natural logarithm; CI: confidence interval; CR: complete revascularization; IR: incomplete revascularization; SD: standard deviation; SYNTAX I: Synergy Between PCI With Taxus and Cardiac Surgery score I.

Supplementary Table 6. Complications due to the coronary intervention

**Variable**	**CR (N=70)**	**IR (N=165)**	***p*-value**
Complications due to coronary intervention, n (%)	3 (4.3)	16 (9.7)	0.199
Malignant arrhythmia	1 (1.4)	4 (2.4)	
Acute stent thrombosis	2 (2.9)	1 (0.6)	
Coronary extravasation	0 (0)	3 (1.8)	
TIMI 0 or 1 flow	0 (0)	3 (1.8)	
Coronary artery dissection	0 (0)	2 (1.2)	
Puncture site complication	0 (0)	3 (1.8)	
Mechanical complications	0 (0)	1 (0.6)	

CR: complete revascularization; IR: incomplete revascularization; TIMI: Thrombolysis In Myocardial Infarction.

Supplementary Figure. *Study flow chart.*

CABG, coronary artery bypass grafting; CR, complete revascularization; CTO, chronic total occlusion; IABP, intra-aortic balloon pump; IR, incomplete revascularization; LAD, left anterior descending coronary artery; MVD: multivessel disease; PCI: percutaneous coronary intervention; STEMI: ST-segment elevation myocardial infarction.

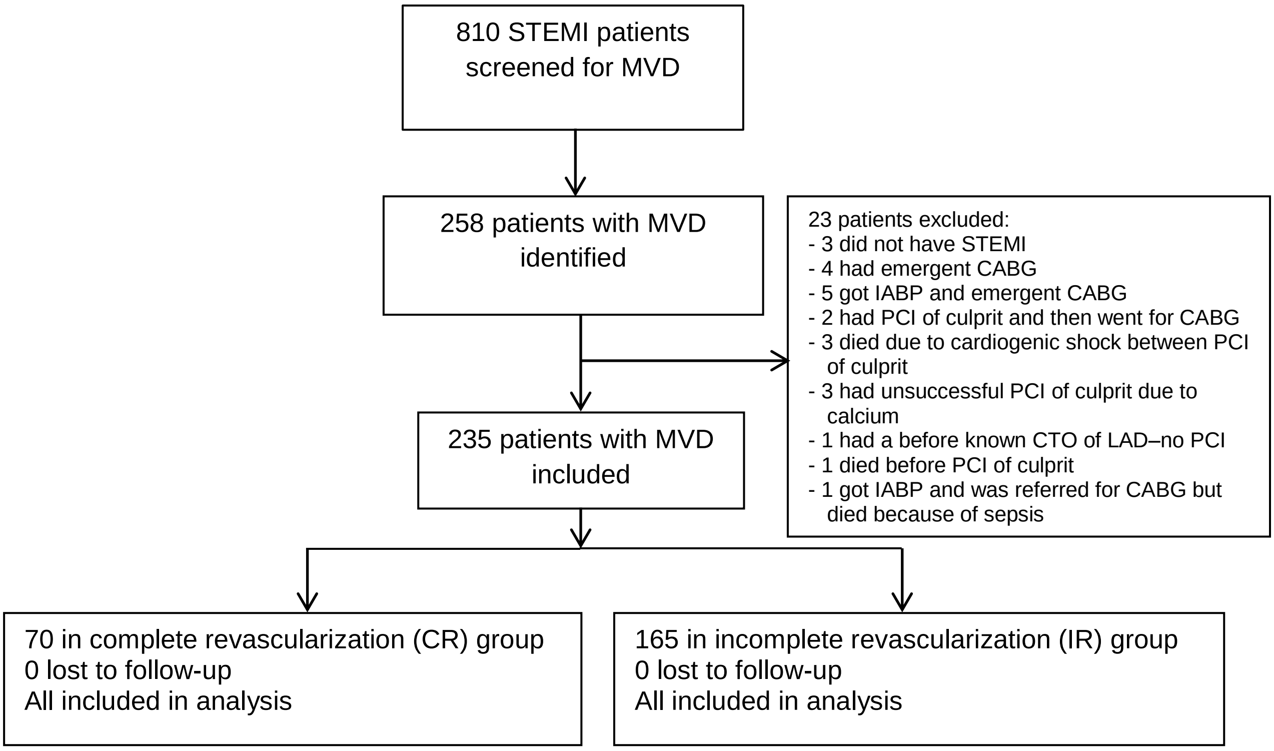

References

1. Fokkema ML, James SK, Albertsson P, Akerblom A, Calais F, Eriksson P, Jensen J, Nilsson T, de Smet BJ, Sjogren I, Thorvinger B, Lagerqvist B. Population trends in percutaneous coronary intervention: 20-year results from the SCAAR (Swedish Coronary Angiography and Angioplasty Registry). *J Am Coll Cardiol* 2013;61:1222-30.
2. Pedersen F, Butrymovich V, Kelbaek H, Wachtell K, Helqvist S, Kastrup J, Holmvang L, Clemmensen P, Engstrom T, Grande P, Saunamaki K, Jorgensen E. Short- and long-term cause of death in patients treated with primary PCI for STEMI. *J Am Coll Cardiol* 2014;64:2101-8.
